# Supplementary material for: Rapid Multianalyte Microfluidic Homogeneous Immunoassay on Electrokinetically Driven Beads
Source: Biosensors (Basel). 2020 Dec 21;10(12):212. doi: 10.3390/bios10120212 (PMC7766682; doi:10.3390/bios10120212)
Supplement: Supplementary file 1 [file biosensors-10-00212-s001.zip › biosensors-1009674 supplementary/2020.12.20 Supplementary DEP Surfing/2020.12.07 Biosensors DEP Surfing SI.docx]

**Supplementary Information: Rapid multianalyte microfluidic homogeneous immunoassay on electrokinetically-driven beads**

Pierre-Emmanuel Thiriet, Danashi Medagoda, Gloria Porro and Carlotta Guiducci


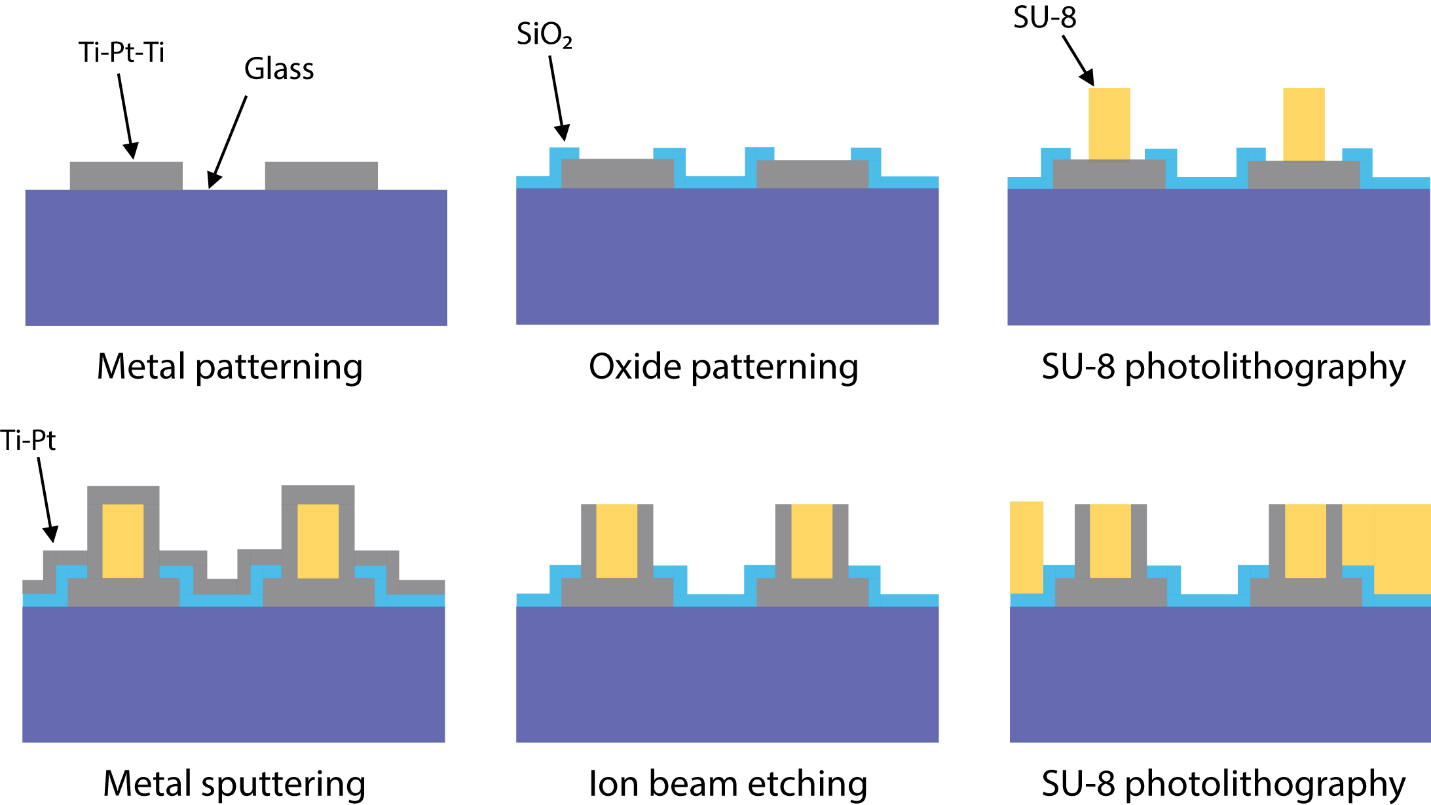


**Figure S1: Fabrication of vertical electrodes in the microfluidic channel**. Schematic illustration of the microfabrication process. First, planar metal lines are partially insulated by silicon dioxide and successively 50 µm-high SU-8 pillars are deposited on top of the open metal regions. Those pillars are then covered with a thin metal layer that is etched everywhere but on the pillars’ walls through vertical etching. The electrical connection between the vertical metal layers on the pillars and the horizontal lines is maintained. Finally, the microfluidic SU-8 channels are patterned through photolithography at a height of 50 µm.


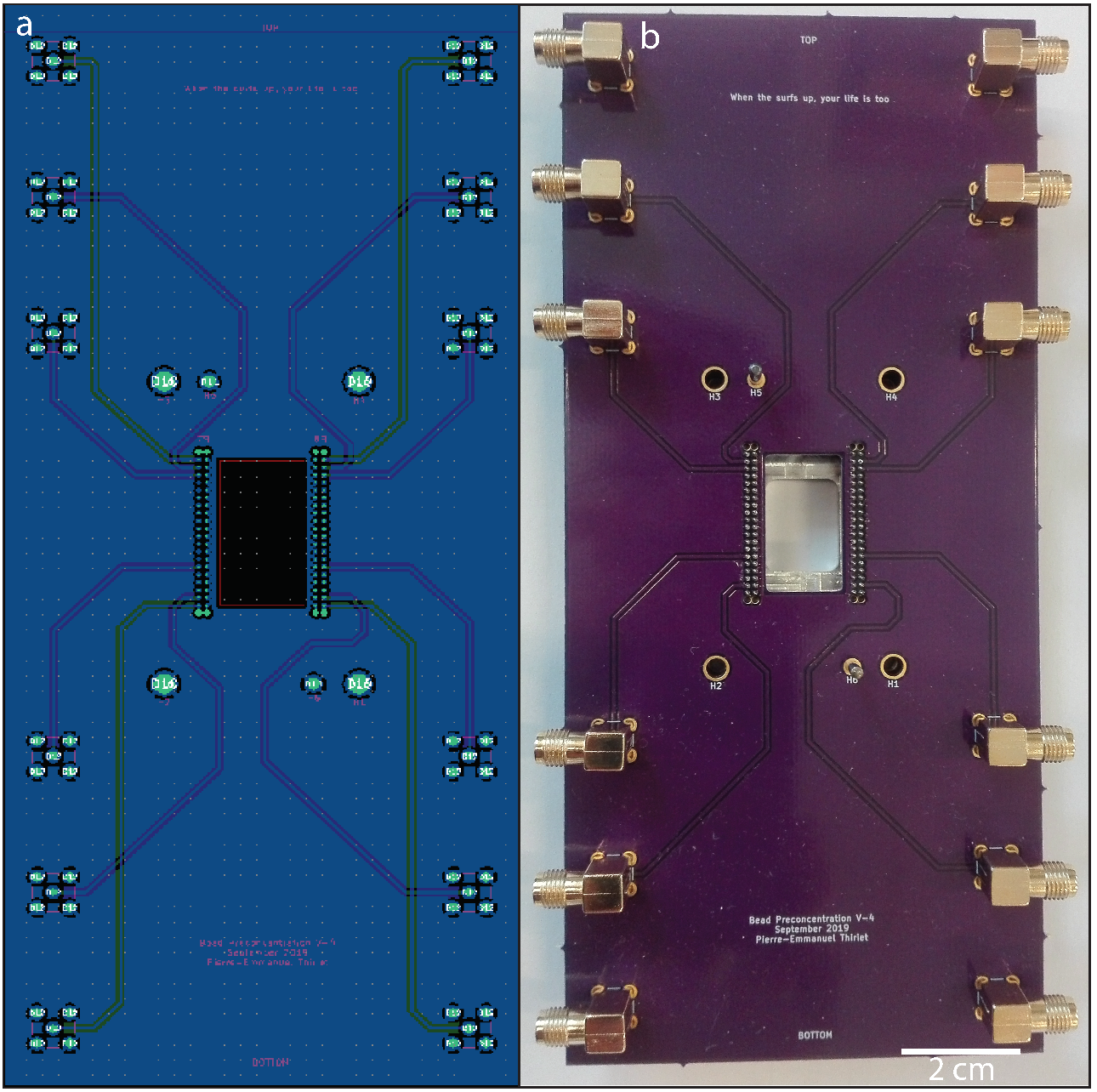


**Figure S2: Design of the PCB.** (a) CAD design of the PCB layout. Copper tracks are shown in blue (top side) and green (bottom side). The tracks bring the signal from the frequency generator to the chip via spring-loaded contacts. (b) Picture of the assembled PCB: the electrical signal for DEP is provided by the six SMA connectors shown on both sides of the PCB.

**
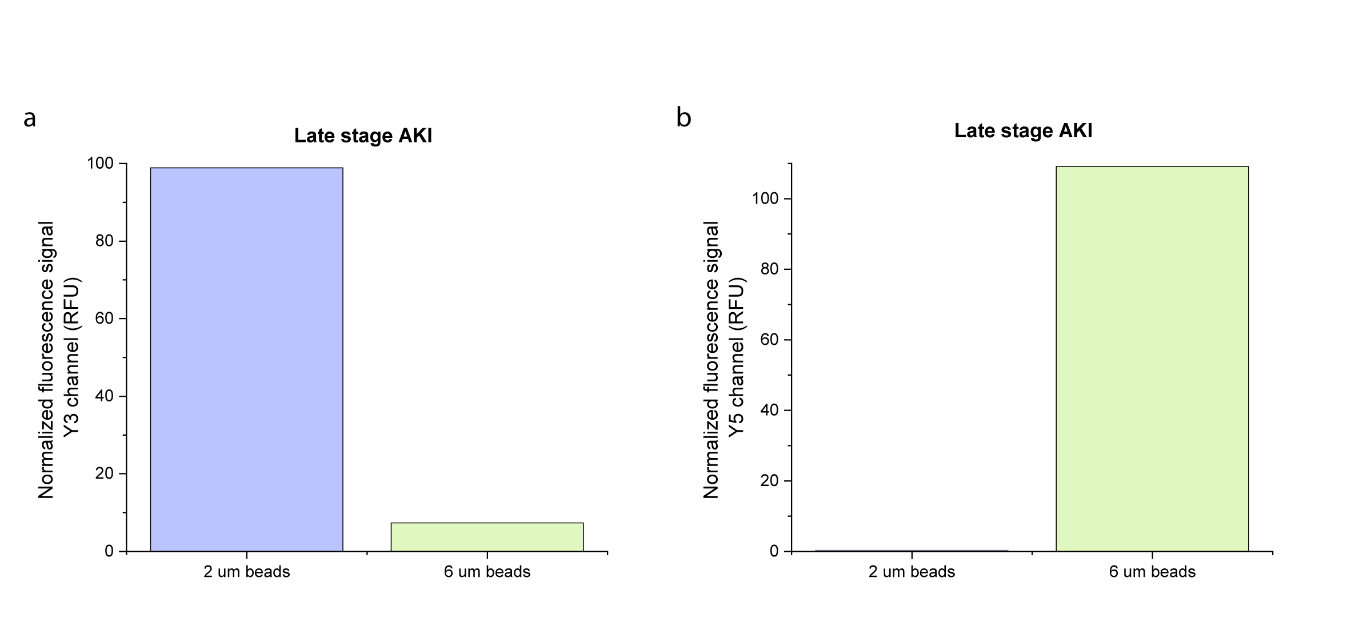
**


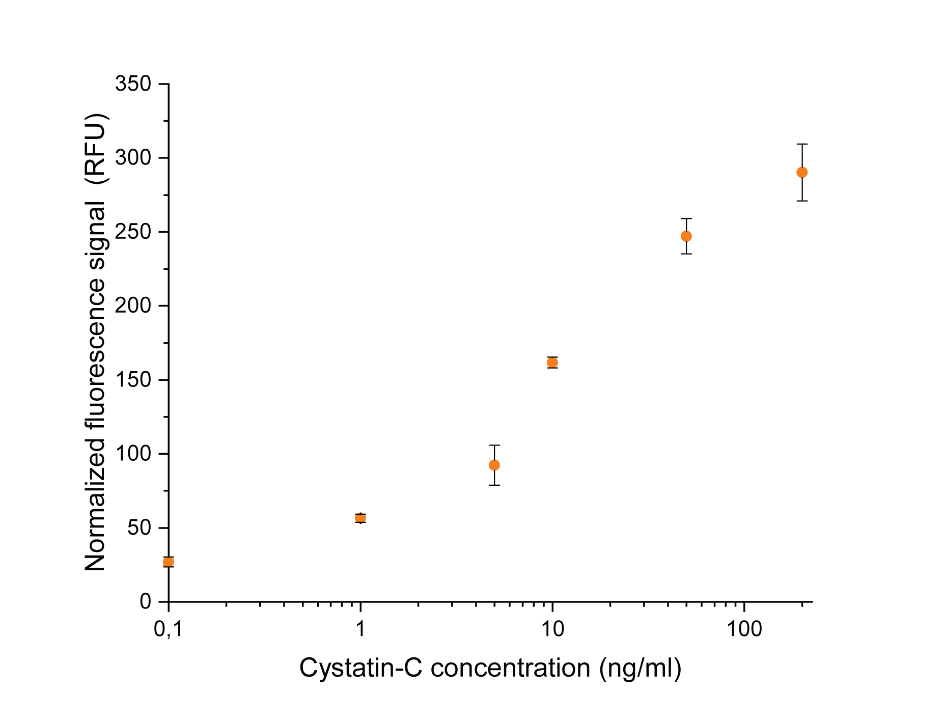
**Figure S3: Spectral overlap of CY3 and CY5 signals from 6 µm NGAL and 2 µm Cystatin C-decorated beads**. Beads were incubated for 15 minutes in vials in the late stage conditions (NGAL concentration: 50 ng/ml, Cystatin C concentration: 1000 ng/ml) and the corresponding signals of single beads were acquired in both CY3 (a) and CY5 (b) fluorescent channels.

**Figure S4: Cystatin C dose response curve.** Results of sandwich immunoassay performed on the microdevice on Cystatin C samples. The flow rate during the incubation is set at 0.4 µL/min and the beads used for this experiment have a diameter of 2 µm. The same molecule was measured also in a multi-analyte configuration (Figure 6). In the present plot, the measured intensities are lower, which could be explained by a decrease of the fluorophore performance due to aging. Error bars were calculated over three measurements. For more details regarding data treatment please refer to *Data analysis* under the *Materials and methods* section.


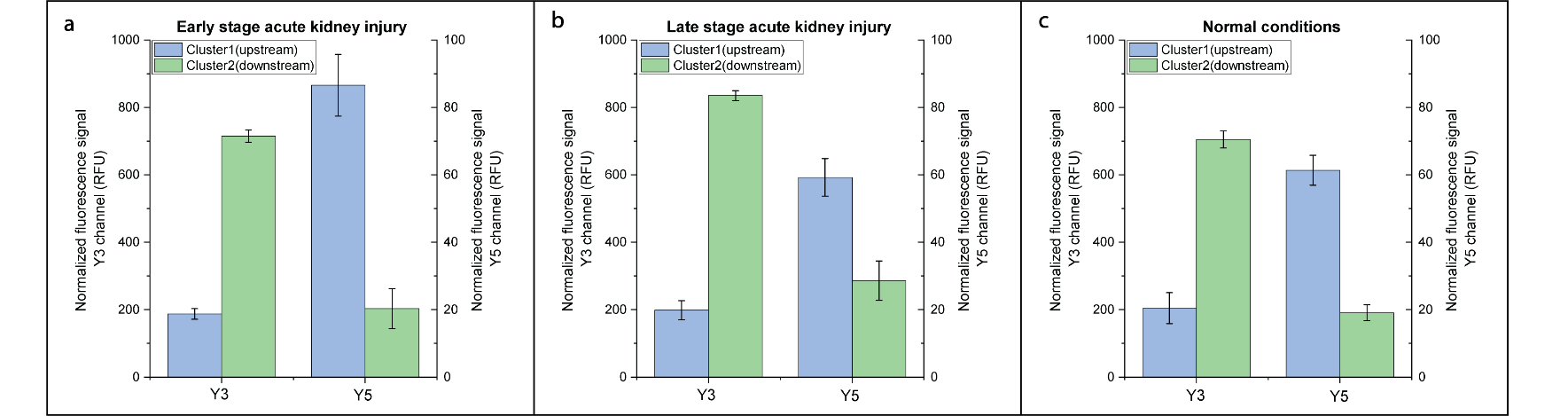



**Figure S5: Cross-contamination analysis in the multianalyte experiment.** Fluorescence signal acquired for both CY5 channel (NGAL detection) and CY3 channel (Cystatin C detection) in cluster 1 (upstream, red cluster in Figure 6a, accumulating 6 µm beads capturing NGAL) and cluster 2 (downstream, green cluster in Figure 6a accumulating 2 µm beads capturing Cystatin C). The three cases presented here are the same as those described in Figure 6. In order to calculate the error bars, three acquisition were performed.

**Figure S6: NGAL detection performance comparison between our immunoassay on chip and a commercial ELISA kit.** The total assay time is about 4 hours for the ELISA kit (R&D biosystems, UK) versus 15 min with our on chip integrated protocol. In terms of performance both assays can detect concentration ranging from 1 to 100 ng/mL (for 1 ng/mL the signal to noise ratio is 4 for ELISA and 3 for the chip), and comparable sensitivity over that entire range. Error bars were calculated over three measurements.
